# Garlic Revisited: Antimicrobial Activity of Allicin-Containing Garlic Extracts against *Burkholderia cepacia* Complex

**DOI:** 10.1371/journal.pone.0112726

**Published:** 2014-12-01

**Authors:** Daynea Wallock-Richards, Catherine J. Doherty, Lynsey Doherty, David J. Clarke, Marc Place, John R. W. Govan, Dominic J. Campopiano

**Affiliations:** 1 University of Edinburgh Medical School, Little France Crescent, Edinburgh, United Kingdom; 2 EastChem School of Chemistry, University of Edinburgh, West Mains Road, Edinburgh, United Kingdom; Ghent University, Belgium

## Abstract

The antimicrobial activities of garlic and other plant alliums are primarily based on allicin, a thiosulphinate present in crushed garlic bulbs. We set out to determine if pure allicin and aqueous garlic extracts (AGE) exhibit antimicrobial properties against the *Burkholderia cepacia* complex (Bcc), the major bacterial phytopathogen for alliums and an intrinsically multiresistant and life-threatening human pathogen. We prepared an AGE from commercial garlic bulbs and used HPLC to quantify the amount of allicin therein using an aqueous allicin standard (AAS). Initially we determined the minimum inhibitory concentrations (MICs) of the AGE against 38 Bcc isolates; these MICs ranged from 0.5 to 3% (v/v). The antimicrobial activity of pure allicin (AAS) was confirmed by MIC and minimum bactericidal concentration (MBC) assays against a smaller panel of five Bcc isolates; these included three representative strains of the most clinically important species, *B. cenocepacia*. Time kill assays, in the presence of ten times MIC, showed that the bactericidal activity of AGE and AAS against *B. cenocepacia* C6433 correlated with the concentration of allicin. We also used protein mass spectrometry analysis to begin to investigate the possible molecular mechanisms of allicin with a recombinant form of a thiol-dependent peroxiredoxin (BCP, Prx) from *B. cenocepacia*. This revealed that AAS and AGE modifies an essential BCP catalytic cysteine residue and suggests a role for allicin as a general electrophilic reagent that targets protein thiols. To our knowledge, we report the first evidence that allicin and allicin-containing garlic extracts possess inhibitory and bactericidal activities against the Bcc. Present therapeutic options against these life-threatening pathogens are limited; thus, allicin-containing compounds merit investigation as adjuncts to existing antibiotics.

## Introduction

*There are spices and vegetables that can grow*

*Some are under the ground, some grow tall*

*Though they all have their qualities, this you should know*

*That the garlic is best of them all*

Ruthie Gordon, The Garlic Waltz (1980)

**Figure 1:**
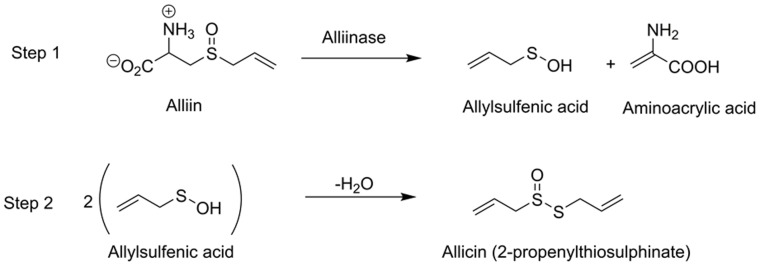
Chemical structure of allicin and mechanism of formation from alliin by the enzyme alliinase. Step 1. Alliinase hydrolyses alliin to produce allylsulfenic acid which, in step 2, condenses spontaneously with the loss of water to produce allicin.

The various medicinal properties of garlic (*Allium sativum* L.) and other alliums have long been recognised; nevertheless, these properties and their *modus operandi* remain enigmatic [1]. The antimicrobial activity derived from alliums was identified nearly 70 years ago and subsequently the chemical structure of allicin (2-propenylthiosulphinate, figure 1) and its properties elucidated over a series of papers by researchers at The Winthrop Chemical Company [2]–[5]. More recent analyses revealed that allicin accounts for approximately 75% of garlic-derived sulphinates [1], [6]–[9]. Amongst over 600 allium species, most attention has been paid to aqueous extracts of garlic which are particularly rich in allicin. In freshly prepared garlic homogenate, allicin is derived *de novo* by the action of the pyridoxal 5′-phosphate-containing enzyme, alliinase, on the non-protein amino acid, alliin (Figure 1) [10]. Unfortunately, the instability of allicin in the presence of other garlic-derived compounds has hampered attempts to distinguish between the antibacterial role of alliin, allicin and other sulfur-rich antibacterial compounds in plant extracts. In addition, most medicinal garlic supplements sold as garlic powder tablets or capsules show poor allicin release [7]. The mechanism(s) through which allicin and other garlic compounds inhibit or kill bacteria also remain unclear. Studies on inhibition of *Salmonella typhimurium* using allicin prepared from reacting alliin with alliin lyase suggested that inhibition of RNA synthesis is a primary target of allicin action [11]. Allicin and other thiosulphinates are also known to react with cysteine to abolish antimicrobial activity [12] and to inhibit acetyl-CoA synthases from plants, yeasts and mammals [13]. A recent review highlights the chemical and biological properties of allicin [14].

Most previous studies of the antibacterial activity of garlic extracts have focused on *Escherichia coli* and *Staphylococcus aureus*. Ajoene, a lipid-soluble allyl sulphide derivative of allicin, found in oil-derived garlic extracts has been reported to cause broad spectrum microbial growth inhibition [15]. Recently, ajoene has also been shown to inhibit quorum sensing in *P. aeruginosa* [16], [17]. Interestingly, we could find no published studies of the antimicrobial properties of garlic-related compounds on the *Burkholderia cepacia* complex (Bcc), a group of 17 closely-related species distributed widely in soil, water and the plant rhizosphere [18]. This is both surprising and ironic since as well as being important agents for bioremediation and biological control [19], [20] the Bcc are the major phytopathogens for allium species [21].

In the last few decades, the Bcc have also emerged as important opportunistic human pathogens, in particular as a cause of life-threatening lung infections in individuals with cystic fibrosis (CF) and chronic granulomatous disease [22], [23]. Although patient segregation and strict infection control have reduced the incidence of Bcc infections in individuals with CF, such infections remain an important clinical problem. At present, the most predominant Bcc species responsible for CF infections are *B. cenocepacia* and *B. multivorans* [22], [24]. Most transplant centres worldwide exclude individuals infected by *B. cenocepacia* from access to lung transplantation, the only proven treatment for severe CF lung disease. Thus, any new strategies that lead to the improved eradication of Bcc from an infected patient would be important. Unfortunately, a common feature of the Bcc is intrinsic resistance to most antibiotics [25]; hence antibiotic treatment presents a major challenge. To our knowledge, there have been only five case reports of successful antibiotic therapy for cepacia syndrome, the acute potentially fatal septicaemia and necrotising pneumonia caused by Bcc. These reports emphasise the need for prolonged treatment with IV and aerosolised antibiotic combinations which include ceftazidime, ciprofloxacin, tobramycin, temocillin and trimethoprim-sulphamethoxazole [26], [27]. At present, there is insufficient data to support the use of any specific antibiotic regimen against Bcc infection [28], [29]. There is an even greater need to have alternative therapies with the recent alarming report of a clinical trial of inhaled aztreonam that failed to treat CF patients with *Burkholderia spp.* infection [30], [31]. Thus, the availability of novel antimicrobial agents against Bcc would represent a major clinical advance for patients with Bcc infection.

Given the dearth of new antibiotics from conventional approaches within the pharmaceutical industry, the development of novel antimicrobial compounds derived from natural sources would be welcomed [32], [33]; this is particularly relevant to the management of lung infections which are the primary cause of morbidity and mortality in the CF population. As previously indicated, the therapeutic potential and complex chemistry of alliums is well documented but remains enigmatic [1], [6]. In our study, we have employed a multidisciplinary approach using both analytical chemistry and microbiology to investigate the antimicrobial properties of a pure aqueous allicin standard (AAS) and aqueous garlic extracts (AGE). Our data demonstrates that the antimicrobial potential of allicin-containing plant extracts is worth revisiting.

## Materials and Methods

### Quantitative RP-HPLC analyses

Allicin standard was purchased from LKT Laboratories, Minnesota as a preparation with 98% purity and was stored at −80°C until used. AGE preparations were prepared from 30 g crushed garlic bulb and 30 ml sterile, distilled water as described by Fujisawa *et. al.* 2008 [6]. We used a method based on that published by Lawson and Wang to analyse the allicin content in the AAS and AGE [7]. We prepared a 1000 µg/ml AAS stock solution and carried out serial dilutions to obtain standards that ranged from 8–1000 µg/mL to calibrate the RP-HPLC method (Column dimensions: 250×4.6 mm, 3.6 µ, C18 Aeris Peptide from Phenomenex). The elution conditions used were: isocratic elution with MeOH/water (60∶40, v/v) at 1 ml/min with UV detection at 240 nm, 25°C. An injection volume of 20 µL was used for all samples. The mass of the AAS was confirmed using direct infusion mass spectrometry. Typically, AAS was analysed at a concentration of 10 µM from a solution of water/acetonitrile (50∶50, v/v). Spectral data was collected on an electrospray micro-ToF mass spectrometer (Bruker) operating in the positive mode.

### Antimicrobial assays

In a preliminary survey, we investigated the minimum inhibitory concentrations (MICs) of AGE by agar dilution against 38 Bcc isolates representing nine Bcc species and which included 30 isolates from an experimental Bcc strain panel [34]. The MICs and minimum bactericidal concentrations (MBCs) of allicin standard (AAS) against five members of the BCC strain panel were determined by the microtitre broth technique recommended by the NCCLS [35]. Time kill assays were then performed on a single Bcc isolate, *B. cenocepacia* C6433, with an initial bacterial inoculum of 10^6^ CFU/mL and with AGE and AAS at concentrations ten times MICs. To provide further information on suitable screening assays, susceptibility to AGE was investigated by agar well diffusion and impregnated paper disc diffusion analysis [6]. Isosensitest agar (Oxoid Ltd., Basingstoke, UK) plates were flood seeded using a log phase 10^6^ CFU/mL isosensitest broth culture of C6433. 10 µL undiluted AGE was added to a well cut in the agar or used to impregnate sterile paper discs. Antimicrobial activity was determined by a zone of inhibition after overnight incubation at 37°C (Figure S1).

### Modification of recombinant *B. cenocepacia* BCP by AAS and AGE

The His-tagged *B. cenocepacia* J2315 bacterioferritin comigratory protein (BCP) peroxiredoxin (Prx), which is also referred to as “peroxidase”, was overexpressed in *E. coli* and purified and stored as described by Clarke *et. al.* [36] The purified BCP protein (10 µM) was incubated with AAS (0.1 mM and 1 mM) at 37°C for 1, 5, 30, 60, 120 and 180 mins. Each reaction was quenched with 0.1% formic acid and then analysed by electrospray ionisation Fourier transform ion cyclotron resonance mass spectrometry (ESI FT-ICR MS). Freshly-prepared AGE (3.38 mg/mL was passed through a PD-10 (Sephadex G-25M) desalting column, the low molecular weight fraction was collected and incubated with BCP (50 µM) for 1, 5, 30, 60, 120 and 180 mins at 37°C. To isolate the enzyme from the incubation mixture, the sample was then shaken with Ni-NTA resin for 50 mins followed by elution of the protein from the resin with 300 mM imidazole. Excess imidazole was removed from protein samples by PD-10 desalting which were then concentrated to 2 mL and analysed by ESI FT-ICR MS.

### Protein Mass Spectrometry Analysis

Protein samples were analysed by LC-MS using an Ultimate 3000 HPLC system (Dionex Corporation, Sunnyvale, CA), equipped with a monolithic PS-DVB (500 µm×5 mm) analytical column (Dionex Corporation). Mobile phases A and B comprised 2∶97.95 and 80∶19.95 acetonitrile∶water with 0.05% formic acid respectively (v/v/v). Samples were injected onto the analytical column, washed with buffer A for 5 min, followed by a 20 min linear gradient elution (20 µl/min) into buffer B. MS data was acquired on a Bruker 12 Tesla Apex Qe FT-ICR (Bruker Daltonics, Billerica, MA). Desolvated ions were detected between *m/z* 600 and 2000 for 0.5 s to yield a broadband 512 Kword time-domain data. Fast Fourier Transforms and subsequent analyses were performed using DataAnalysis (Bruker Daltonics) software. All *m/z* spectra were deconvoluted using MaxEnt software in DataAnalysis (Bruker Daltonics).

## Results

### Quantification of allicin in AGE

**Figure 2:**
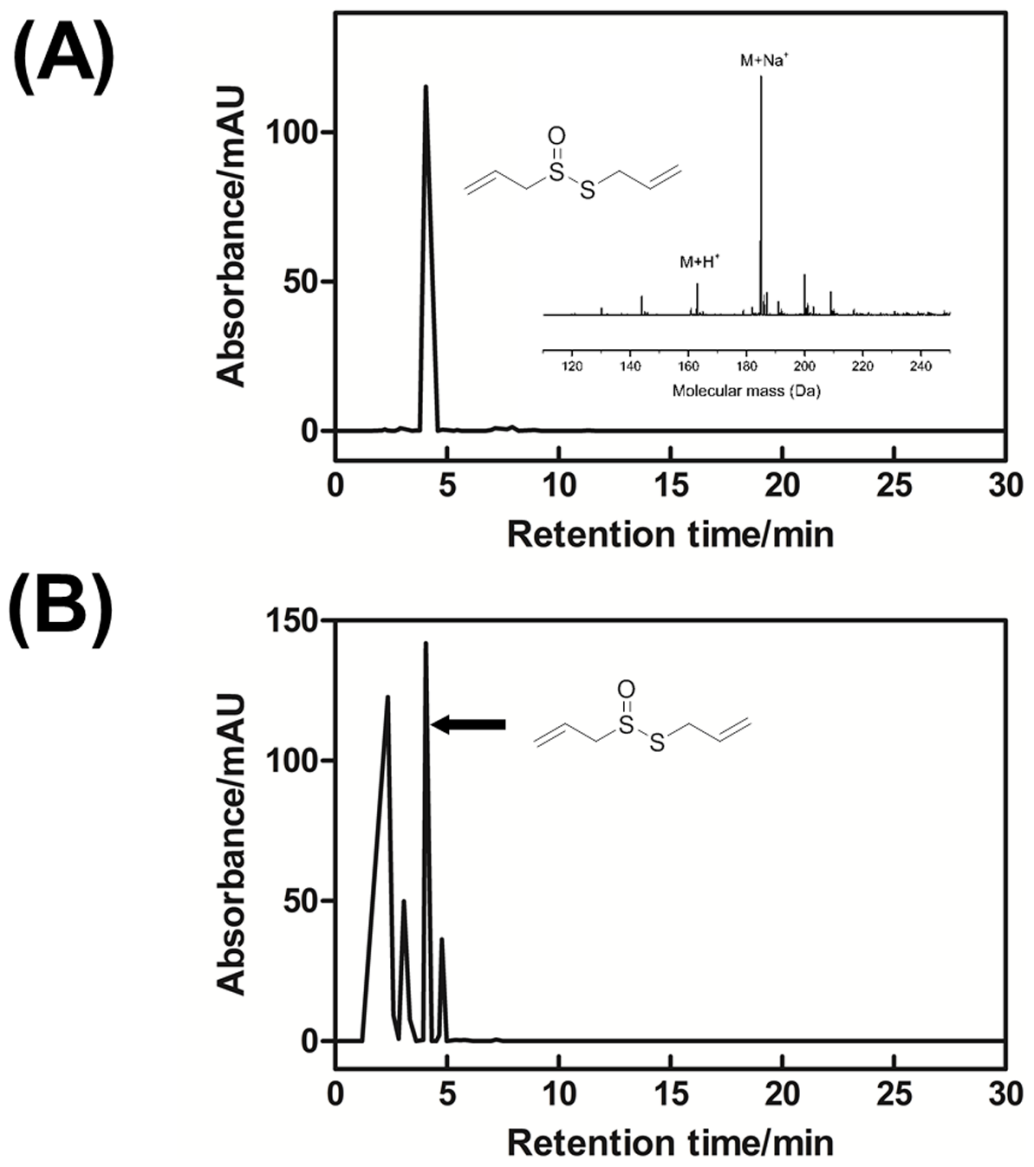
HPLC and MS analysis of aqueous allicin standard (AAS) and aqueous garlic extract (AGE). (A) AAS was analysed using a C18 reverse phase column with UV detection at 240 nm. The standard eluted at 4.1 minutes. AAS had an observed *m/z* of 185.0067 (consistent with the [M+Na]^+^ species; theoretical *m/z* 185.0065; [C_6_H_10_OS_2_+Na]^+^) (*inset*). (B) AGE was analysed by the same method as described for AAS. The peak at 4.1 minutes corresponds to the mass of allicin.

**Figure 3:**
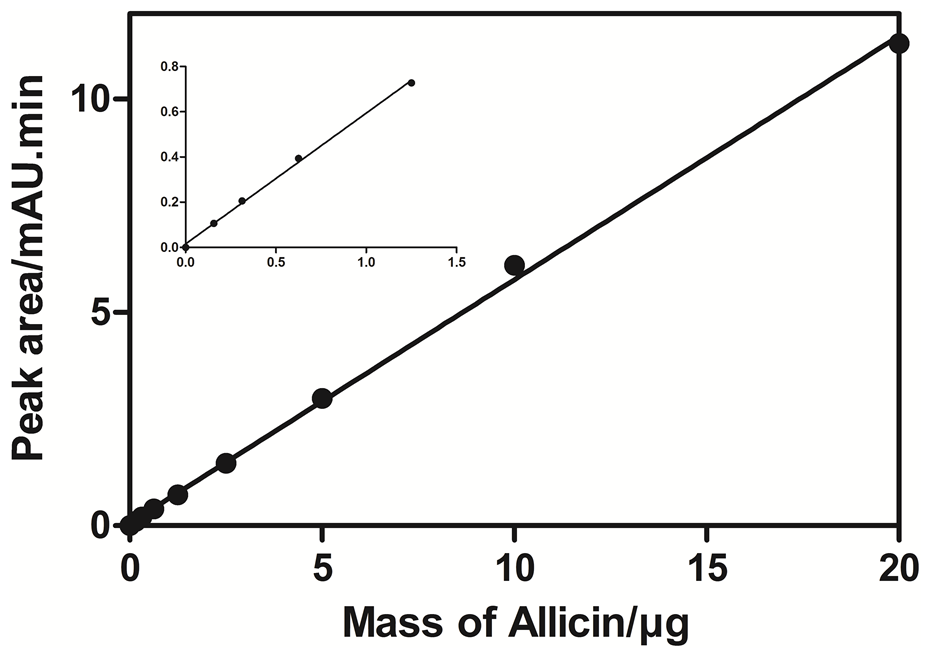
Calibration curve of aqueous allicin standard (AAS). AAS of known concentrations (8–1000 µg/mL) were used to produce a calibration curve which was then used to quantify the allicin present in the fresh AGE preparations. The peak area for allicin was linear over 0–20 µg of allicin loaded onto the column (r^2^ = 0.9987 and gradient 0.57±0.01 mAU.min. µg^−1^).

The purity of AAS was confirmed using a published RP-HPLC method (Fig. 2A) [7]. Allicin eluted with a retention time of 4.1 min and an observed *m/z* of 185.0067 (consistent with the [M+Na]^+^ species; theoretical *m/z* 185.0065; [C_6_H_10_OS_2_+Na]^+^) (Fig. 2A inset). Using the same HPLC conditions, the elution profile of AGE contained several well-resolved peaks (Fig. 2B), and the peak eluting at 4.1 min was identified as allicin by mass spectrometry (*m/z* 185.0067; [M+Na]^+^). A calibration curve was obtained by analysing known concentrations of AAS (8–1000 µg/mL) and used to quantify the amount of allicin present in the fresh AGE preparations (Fig. 3). The peak area for allicin was linear over 0–20 g of allicin loaded onto the column (r^2^ = 0.9987 and gradient 0.57±0.01 mAU.min. µg^−1^) and the amount of allicin in freshly prepared AGE was determined as 67.6±7.9 µg in 20 µl, which equates to 3.38×10^3^±0.39 µg/mL (allicin/AGE, w/v).

### Antimicrobial activity

AGE displayed antimicrobial activity against all of the 38 Bcc isolates (Table 1) with MICs ranging from 0.5 to 3%, where 100% equates to 10 g of garlic bulb homogenised in 10 ml of sterile, distilled water. Interestingly, comparison of the antimicrobial activity of four independent AGE preparations showed little batch-to-batch variation (data not shown). Activity also remained stable when AGE was stored at 4°C for seven days or for at least 6 months at −80°C. We noted that the epidemic, clinically-relevant isolate *B. cenocepacia* C6433 was sensitive (0.5%) to AGE. So in further experiments, the inhibitory activity of AGE was confirmed with impregnated paper discs and agar well diffusion assays using an inoculum of 10 µl (10^6^ CFU/ml) per disc or well (Figure S1). Since the amount of pure allicin (AAS) available was limited, investigation of the antimicrobial activity of AAS was restricted to MIC and MBC assays using five Bcc isolates (table 2). MICs ranged from 8 to 62 µg/mL and MBCs 31 to 62 µg/mL. In time-kill assays, bactericidal activities of AGE and AAS against *B. cenocepacia* C6433 were 99.9% and 100% respectively within 12 h (data not shown). Since the MIC of AGE required for inhibition of *B. cenocepacia* C6433 is 0.5%, we used our quantification of allicin in garlic preparations (Fig. 3) to determine that this dilution of AGE contains ∼16.9 µg/mL allicin. This analysis now allows comparison with the MIC of pure allicin (AAS) against this *B. cenocepacia* isolate, which we determined as 4 µg/mL allicin. These observations suggest that, against this isolate, allicin is the major antibacterial species in the AGE preparation.

**Table 1:** MICs (µg/ml) of AGE for *B. cepacia* complex.

Strain	Edinburgh Lab No.	LMG No.	*Strain Name	AGE (%)
*B. cepacia*	J673	1222	ATCC 25416	2
*B. cepacia*	J675	2161	ATCC 2161	1
*B. cepacia*	C2970	17997		0.5
*B. cepacia*	C3159	18821	CEP509	0.5
*B. multivorans*	C1576	16660		0.5
*B. multivorans*	C1962	16665		0.5
*B. multivorans*	C3160	18822	C5393	1
*B. multivorans*	C3161	13010		1
*B. multivorans*	C3162	18825	CF-A1-1	1
*B. multivorans*	C3163	18824	JTC	0.5
*B. multivorans*	C3164	18823	249-2	0.5
*B. multivorans*	J2511	17588	ATCC 17616	1
*B. cenocepacia*	J415	16654		1
*B. cenocepacia*	C1394	16659		1
*B. cenocepacia*	J2315	16656		0.5
*B. cenocepacia*	J2524	18832	ATCC 17765	3
*B. cenocepacia*	C3165	18826	BC7	0.5
*B. cenocepacia*	C3166	18863	K56-2	1
*B. cenocepacia*	C3167	18827	C5424	0.5
*B. cenocepacia*	C3168	18828	C6433	0.5
*B. cenocepacia*	C3169	18829	PC184	0.5
*B. cenocepacia*	C3170	18830	CEP511	1
*B. stabilis*	C3171	14294		0.5
*B. stabilis*	C3172	18870	C7322	0.5
*B. stabilis*	C3173	18888		1
*B. stabilis*	C3174	14086		1
*B. vietnamiensis*	C2978	16232		1
*B. vietnamiensis*	C3175	18835	PC259	1
*B. vietnamiensis*	C3176	18836	FC441	3
*B. vietnamiensis*	C3177	10929		1
*B. dolosa*	J3357			1
*B. dolosa*	J3358			1
*B. ambifaria*	J3361			2
*B. ambifaria*	J3362			1
*B. anthina*	J3364			1
*B. anthina*	J3366			1
*B. pyrrocinia*	J3368			2
*B. pyrrocinia*	J3369			0.5

* **We have included all codes to avoid confusion between Edinburgh strain collection numbers, LMG and ATCC codes.**

**Table 2:** MICs and MBCs (µg/ml) of AAS for *B. cepacia* complex.

Strain	Edinburgh Lab No.	LMG No.	MIC	MBC
*B. cenocepacia*	J2315	16656	8	31
*B. cenocepacia*	C6433	18828	8	31
*B. cenocepacia*	C3165	18826	16	31
*B. stabilis*	C3172	18870	16	31
*B. cepacia*	J673	1222	62	62

### Interaction of BCP with allicin and AGE

**Figure 4:**
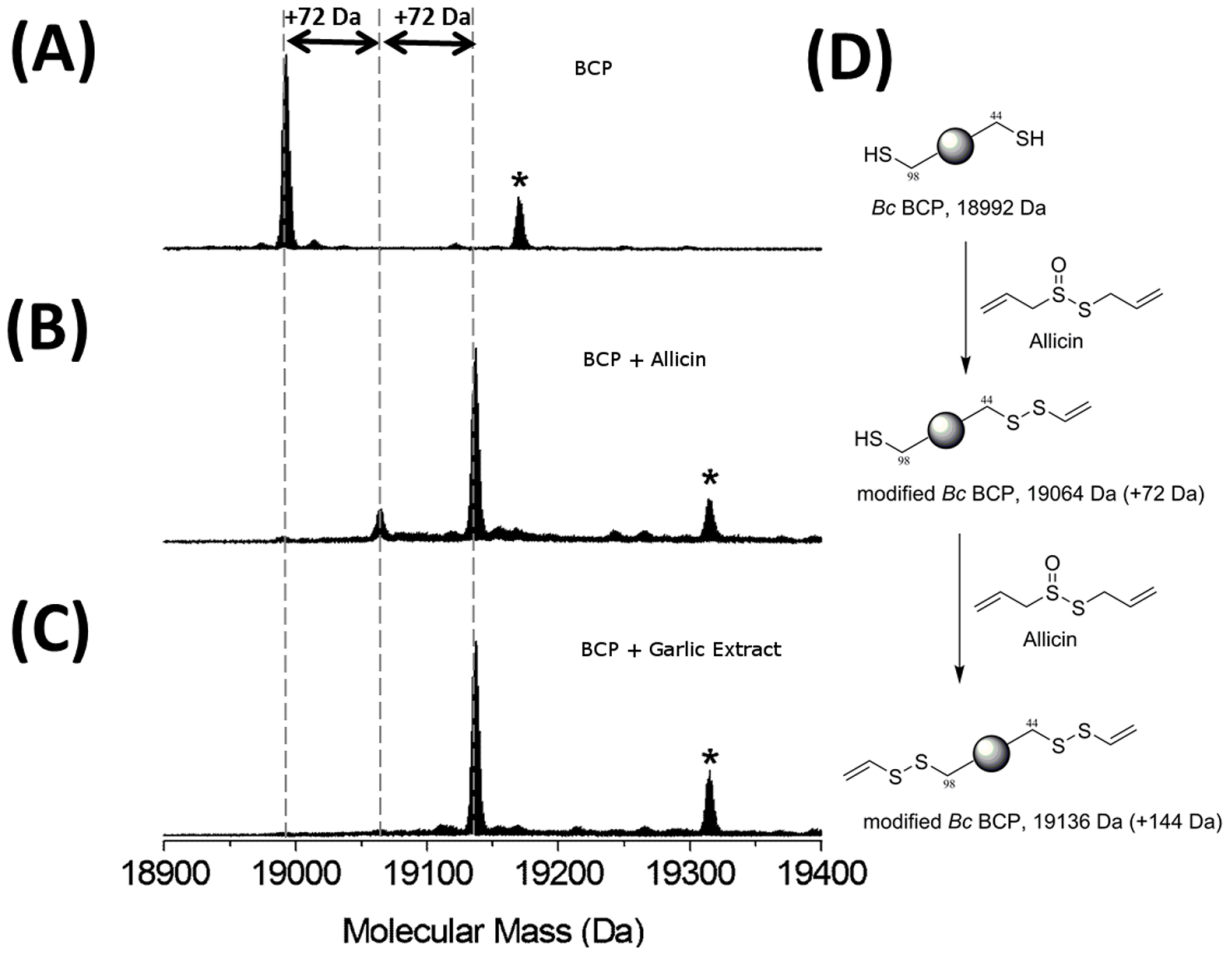
MS analysis of recombinantly produced *Burkholderia cepacia* BCP Prx incubated with AAS and AGE. (A) Purified *Bc* BCP Prx has a mass of 18992 Da, consistent with its predicted mass based on the amino acid sequence (B) *Bc* BCP after incubation with 1 mM AAS for 1 h results in the formation of two covalently modified species of mass increases +72 Da and +144 Da. (C) *Bc* BCP Prx after incubation with AGE for 1 h results in the formation of a covalently modified species of mass increase +144 Da corresponding to the addition of two allicin adducts. * denotes enzyme species that undergo α-N-gluconoylation during protein expression. (D) Proposed mechanism of reaction of allicin with *Bc* BCP Prx.

The exact chemical mechanism(s) by which allicin exerts its antibacterial activity has been the subject of numerous studies and it's chemical structure suggests it should react with free thiols. Indeed, allicin is known to covalently modify the free amino acid L-cysteine via formation of allyl-disulfide species [37]. Therefore, we postulate that the antimicrobial activity of allicin is derived from modification of cysteine residues found in key metabolic proteins within *Burkholderia*. In order to test this hypothesis we have studied the reaction of AAS and AGE with the enzyme BCP, a peroxiredoxin (Prx) which is known to contain catalytically important cysteine residues (Cys44 and Cys98) [36]. We use this protein as an example of cysteine-containing proteins within the *B. cenocepacia* proteome and not as the sole target of allicin within the organism. The interaction between AAS and the recombinant BCP was studied by mass spectrometry. The unmodified BCP has a mass of 18992 Da which confirmed that both cysteine residues are in the reduced (-SH) form (Fig. 4A). Upon incubation of the BCP with AAS (1 mM or 161 µg/ml) for 60 minutes we noted the appearance of two new species with masses of 19064 and 19136 Da (Fig. 4B). These observed increases in mass (Δ72 and Δ144 Da) are consistent with the addition of a single allyl thiol group (C_3_H_5_S) to either one or both cysteine residues (Fig. 4D). This result is not surprising as allicin is expected to react with the free thiols on BCP in a non-specific manner as shown by previous studies with cysteine, glutathione and papain [12], [37]. Importantly, similar mass increases were observed when we incubated the BCP with AGE for 60 minutes (Fig. 4C). This important experiment confirms that the AGE and the AAS react with the protein target in the same manner (Fig. 4D).

## Discussion

Production of antimicrobial compounds by plant alliums which target *Burkholderia* species, their principal bacterial pathogen, is not surprising. However, what is surprising is that, to our knowledge; such activity has not been reported or investigated previously. We report the first evidence for the antimicrobial activity of allicin against the Bcc. The MICs and MBCs of allicin-containing preparations against BCC isolates were commensurate with breakpoint values for antibiotics in present clinical use. Encouragingly, killing activity includes *B. cenocepacia*, the most prevalent and transmissible Bcc species isolated from CF infections [22], [24]. In general, Bcc isolates exhibit intrinsic resistance to antimicrobial peptides and other antibiotics [25], [38], [39]. Thus, treatment of Bcc infections remains challenging and no particular antibiotic regimen is presently recognised [26], [40].

The chemical mechanisms involved in the bactericidal action of allicin are poorly understood, and in the case of the Bcc are unknown. Given previous evidence that allicin preferentially interacts with cysteine, our hypothesis is that the allylthio moiety of allicin allows it to react with cysteine-containing *Burkholderia* enzymes involved in key biosynthetic pathways. The exact targets of allicin are not known but we assume there will be many proteins that have the potential to be modified. As a relevant model protein, we used the Bcc-derived peroxidase enzyme, BCP peroxiredoxin, which we have previously shown plays a role in the detoxification of reactive oxygen species (ROS) within *B. cenocepacia* [36]. In that study we also identified that Cys44 and Cys98 are essential for the optimal catalytic activity of the enzyme, therefore any chemical modification of these residues will inactivate the enzyme. We chose mass spectrometry as the analytical tool for BCP/allicin analysis since BCP ionises well and mass spectrometry is ideally suited to measure covalently-bound adducts.

Our data strongly suggests that pure allicin (AAS) and, more importantly, the allicin within AGE react with both Cys44 and Cys98 residues of BCP to give modified isoforms of masses 19064 Da and 19136 Da. These observed mass increases of 72 and 144 Da correspond to the addition of one and two allylthio groups respectively by reaction with allicin (Fig. 4D). The proposed mechanism for the formation of the BCP-allythio derivative involves nucleophilic attack of a BCP cysteine thiol (either Cys44 or Cys98) on the more electrophilic sulfur of allicin to produce a new disulfide bond. A second molecule of allicin undergoes a similar reaction to add a second allylthio group. Reduction of the derivitised BCP with excess reducing agent (*tris* (2-carboxyethyl) phosphine, TCEP) returned the enzyme to the unmodified form (18992 Da) which suggests that the reaction of allicin with BCP is chemically reversible in the presence of strong reducing agents (data not shown). The results from the proteomic analysis of BCP with the AGE was very unexpected, as garlic extract is a complex mixture as observed from our HPLC analysis. This clearly-resolved data confirms that allicin is the main component of freshly prepared garlic that reacted with BCP (Fig. 4C). More importantly, this is the first study where AAS and AGE have been analysed by protein mass spectrometry with a relevant protein target from a bacterial pathogen. We think that this study opens the door to apply this powerful analytical tool for identifying many of the protein targets that are modified by allicin within the proteome of *Burkholderia* and other pthogens. It could also prove useful to study other similar allylthio-containing natural products (e.g. ajoene) derived from allicin.

Previous investigations of novel antimicrobial compounds against Bcc species have included mushroom extracts [41], polyketides [42], docosahexaenoic omega- 3 fatty acid [43] and microbe-derived volatile organic compounds [44]. Our results demonstrating the inhibitory activity of allicin-containing garlic extracts against *Burkholderia* species provide another potential line of research that is particularly relevant to Bcc infection. Evidence from animal and human studies have shown that allicin-containing garlic homogenates, delivered orally, intravenously or intraperitoneally, act as potent antioxidants leading to reduction of neutrophil-mediated lung damage, a major mechanism of Bcc lung infection [45]. The stability of the antimicrobial properties of AGE during storage at 4°C and −80°C coupled with the batch-to-batch reproducibility were notable. Similarly, the rapid therapeutic action of garlic homogenates in serum and plasma in the treatment of cardiovascular disorders [46], [47] could be a particular advantage in the treatment of the life-threatening septicaemia that is characteristic of cepacia syndrome. Our investigation suggests that further work is required to comprehensively describe the antimicrobial mechanisms of allicin and to assess allicin-containing formulations as adjuncts to conventional antimicrobial agents presently used against this challenging group of bacterial pathogens.

## Supporting Information

Figure S1 **Zones of inhibition against** ***B. cenocepacia*** **C6433 produced by AGE-impregnated disc (left) and AGE-containing agar well (right).** Isosensitest agar plates were flood seeded using a 10^6^ CFU/mL isosensitest broth culture of C6433. 10 µL undiluted AGE was added to a well cut in the agar or used to impregnate sterile paper discs. We note the lack of resistant mutants.
(TIF)
