# Supplementary figures and images for: Garlic Revisited: Antimicrobial Activity of Allicin-Containing Garlic Extracts against Burkholderia cepacia Complex
Source: PLoS One. 2014 Dec 1;9(12):e112726. doi: 10.1371/journal.pone.0112726 (PMC4249831; doi:10.1371/journal.pone.0112726)

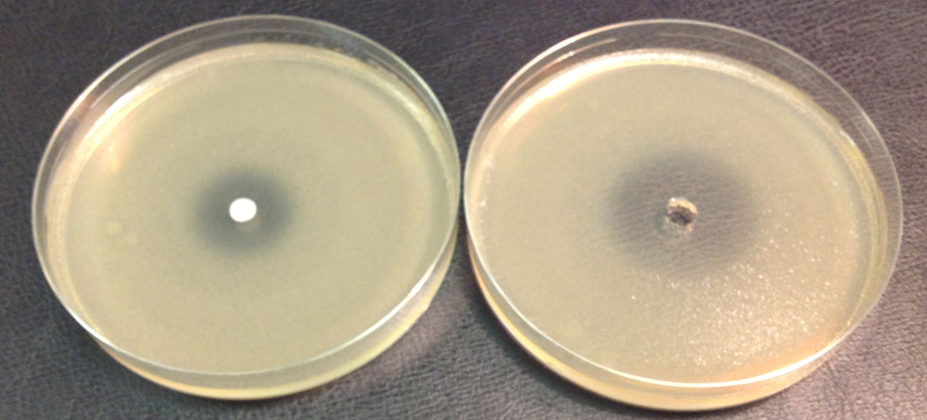

Supplement: Figure S1 — Zones of inhibition against B. cenocepacia C6433 produced by AGE-impregnated disc (left) and AGE-containing agar well (right). Isosensitest agar plates were flood seeded using a 106 CFU/mL isosensitest broth culture of C6433. 10 µL undiluted AGE was added to a well cut in the agar or used to impregnate sterile paper discs. We note the lack of resistant mutants. (TIF) [file pone.0112726.s001.tif]
